# Editorial: Antioxidants from Food and Food Wastes for Nutraceutical, Pharmaceutical and Cosmetic Fields

**DOI:** 10.3390/antiox15020226

**Published:** 2026-02-09

**Authors:** Vincenzo Disca, Monica Locatelli

**Affiliations:** 1 Department of Pharmaceutical Sciences, Università del Piemonte Orientale, Largo Donegani 2, 28100 Novara, Italy; 2 nutractiva Srl, Largo Donegani 2, 28100 Novara, Italy

Antioxidants from foods and agri-food by-products are becoming increasingly recognized as strategically important for the transition toward greener, circular value chains in the nutraceutical, pharmaceutical, and cosmetic sectors. The scientific challenge is no longer limited to demonstrating antioxidant capacity, per se, but rather to building robust pipelines that connect (i) sustainable recovery and “green” extraction, (ii) advanced chemical characterization and standardization, and (iii) formulation strategies that preserve stability and improve bioavailability, enabling applications that are clinically and industrially relevant. The ten contributions published in this Special Issue collectively address these priorities, spanning from marine and terrestrial matrices to extraction and process intensification, chemical fingerprinting, and translational endpoints in metabolic health and skin aging.

A core pillar of this Special Issue is the concern with sustainable extraction and process optimization, with several studies explicitly tackling how recovery technologies can shape yield, composition, and antioxidant performance. Tapia-Quirós et al. (2025) focus their study on olive leaves, an abundant agricultural residue, and illustrate how the valorization of this widely available by-product can be aligned with functional ingredient design (Contribution 1).

In the same “process-to-function” logic, Nabiyeva et al. (2025) optimize conventional and ultrasound-assisted extraction to maximize the total phenolic recovery and in vitro antioxidant activity from *Crataegus almaatensis* leaves, exemplifying how extraction intensification can be used as a lever to increase bioactivity while supporting more sustainable processing routes (Contribution 2).

Krivošija et al. (2025) similarly address extraction, in their case through using subcritical water and pressurized solvents to explore orange peel “herbal dust” as a source of antioxidant compounds (Contribution 3).

Together, these papers reinforce one of the key messages of this Special Issue: the efficacy of antioxidant ingredients is directly correlated to the technologies used to recover them, and so optimized and scalable extraction methods are prerequisites for reproducible nutraceutical and cosmetic development.

A second pillar of this Special Issue is advanced characterization and analytical rigor, which are essential to overcome the variability inherent in by-products and complex natural matrices. Rivera-Tovar et al. (2025) offer a strong contribution to this objective through an integrated extraction–purification workflow for recovering phlorotannins from *Durvillaea incurvata*, coupled with advanced characterization, an approach that directly addresses the Special Issue’s call for analytical methods that enable meaningful quality control and application-driven purification (Contribution 4).

Extending the omics dimension even further, Martakos et al. (2026) provide a lipidomics-centered case study on marine by-product oils, showing how biological origin and extraction technology can reshape lipid class distribution and antioxidant molecule retention, supporting traceability, standardization, and product development for high-value applications (Contribution 5).

Importantly, the marine focus expands the scope of this Special Issue beyond polyphenol-centric narratives, illustrating how antioxidant innovation from by-products also includes lipid-soluble antioxidants (e.g., tocopherols, carotenoids, squalene) and PUFA-rich matrices that require oxidation-aware processing.

A third major theme is formulation and application-readiness in the cosmetic field, where antioxidant value must translate into measurable skin health outcomes which are relevant to photoaging and inflammation. Chiu et al. (2025) assess djulis (*Chenopodium formosanum*) leaf extract as a cosmetic antioxidant ingredient, representing the pathway from the botanical source to skin-directed application (Contribution 6).

Complementarily, Hyun et al. (2025) move into a higher translational level by validating a synergistic marine nutricosmetic ingredient (GABALAGEN^®^) through randomized human trials, aligning closely with the Special Issue’s emphasis on efficacy and delivery (Contribution 7).

Taken together, these contributions highlight two routes for cosmetic innovation, topical cosmeceutical ingredients derived from plant-based resources and ingestible nutricosmetic approaches derived from marine resources, both of which are dependent on robust characterization and stability-preserving processing.

A fourth pillar involves nutraceutical and health-oriented positioning, particularly for metabolic disorders where oxidative stress and inflammation intersect with diet. Buonaiuto et al. (2025) examine betaine supplementation and its biological relevance in vivo, contributing to the broader discussion of antioxidant nutraceutical strategies that influence redox, inflammatory, and metabolic homeostasis (Contribution 8).

At a broader level, Yuksek et al. (2025) review the use of dietary supplements derived from food by-products in diabetes management, explicitly integrating sustainability with mechanistic plausibility, safety, toxicology, and regulatory considerations, which are precisely the translational bottlenecks highlighted in this Special Issue’s scope (Contribution 9). This review is of particular importance because it frames valorized by-products not only as sources of bioactive compounds, but as candidates that must meet real-world constraints: standardization, contaminant control, dosing reproducibility, and regulatory compliance.

Finally, the Special Issue is anchored by a wide-angle perspective on the polyphenols derived from by-products. Demir et al. (2026) review the polyphenols recovered from by-products, discussing their applications and health effects, and thereby connecting extraction technologies, compound classes, and downstream uses across the nutraceutical, pharmaceutical, and cosmetic spectra (Contribution 10).

This contribution provides a conceptual backbone for the Special Issue: polyphenols remain dominant targets for waste-valorization strategies, but their successful deployment depends on matching recovery methods to compound stability, ensuring compositional control, and designing formulations that preserve activity through processing and storage.

Across all the published works, a cohesive narrative emerges that is well-aligned with the stated aims of this Special Issue. Firstly, by-products are treated as legitimate, high-value feedstocks, not as marginal residues, regardless of whether they are olive leaves, hawthorn leaves, orange peels, macroalgae, or marine processing side streams. Secondly, the methodological choices (extraction, purification, and analytical workflows) are shown to be decisive, often being used to determine whether an “antioxidant ingredient” is reproducible, stable, and application-ready. Third, the collection demonstrates a welcome move toward translation, as is exemplified by the clinical evaluation in nutricosmetics and by the reviews that foreground safety, regulatory frameworks, and real formulation constraints.

Overall, this Special Issue emphasizes the need for harmonized methodologies, improved quality control, and sustainability-oriented strategies that can support the effective translation of antioxidant research. On this basis, a second edition will expand the discussion by concentrating on advanced extraction methods, novel formulation approaches, and sustainable technologies, with the aim of further strengthening the practical implementation of antioxidant systems.

